# Supplementary material for: The effects of resistance training to near volitional failure on motor unit recruitment during neuromuscular fatigue
Source: PeerJ. 2024 Oct 14;12:e18163. doi: 10.7717/peerj.18163 (PMC11485100; doi:10.7717/peerj.18163)
Supplement: Supplemental Information 1 [file peerj-12-18163-s001.docx]

| Week 1 | | | | | | | | |
| --- | --- | --- | --- | --- | --- | --- | --- | --- |
| Day 1 | | | Day 2 | | | Day 3 | | |
| Exercise | Weight | SxR | Exercise | Weight | SxR | Exercise | Weight | SxR |
| Squat | 70% | 3x * | Bench | 70% | 3x * | Deadlift | 70% | 3x * |
| Deadlift | 65% | 3x * | Squat | 65% | 3x * | Bench | 65% | 3x * |
| RFESS | 60% | 3x15 | Low-incline Bench | 60% | 3x15 | OHP | 60% | 3x15 |
| RDL | 60% | 3x15 | Lat Pulldown | 60% | 3x15 | BB Row | 60% | 3x15 |
| Face-pull | 60% | 3x15 | Goblet Squat | 60% | 3x15 | BB curl | 60% | 3x15 |
| Skull Crushers | 60% | 3x15 |  |  |  | Lat Raises | 60% | 3x15 |
| Week 2 | | | | | | | | |
| Day 4 | | | Day 5 | | | Day 6 | | |
| Exercise | Weight | SxR | Exercise | Weight | SxR | Exercise | Weight | SxR |
| Squat | 77.5% | 5x * | Bench | 77.5% | 5x * | Deadlift | 77.5% | 5x * |
| Deadlift | 70% | 3x * | Squat | 70% | 3x * | Bench | 70% | 3x * |
| RFESS | 65% | 3x12 | Low-incline Bench | 65% | 3x12 | OHP | 65% | 3x12 |
| RDL | 65% | 3x12 | Lat Pulldown | 65% | 3x12 | BB Row | 65% | 3x12 |
| Face-pull | 65% | 3x12 | Goblet Squat | 65% | 3x12 | BB curl | 65% | 3x12 |
| Skull Crushers | 65% | 3x12 |  |  |  | Lat Raises | 65% | 3x12 |
| Week 3 | | | | | | | | |
| Day 7 | | | Day 8 | | | Day 9 | | |
| Exercise | Weight | SxR | Exercise | Weight | SxR | Exercise | Weight | SxR |
| Squat | 85% | 4x * | Bench | 85% | 4x * | Deadlift | 85% | 4x * |
| Deadlift | 75% | 3x * | Squat | 75% | 3x * | Bench | 75% | 3x * |
| RFESS | 70% | 3x10 | Low-incline Bench | 70% | 3x10 | OHP | 70% | 3x10 |
| RDL | 70% | 3x10 | Lat Pulldown | 70% | 3x10 | BB Row | 70% | 3x10 |
| Face-pull | 70% | 3x10 | Goblet Squat | 70% | 3x10 | BB curl | 70% | 3x10 |
| Skull Crushers | 70% | 3x10 |  |  |  | Lat Raises | 70% | 3x10 |
| Week 4 | | | | | | | | |
| Day 10 | | | Day 11 | | | Day 12 | | |
| Exercise | Weight | SxR | Exercise | Weight | SxR | Exercise | Weight | SxR |
| Squat | 90% | 5x * | Bench | 90% | 5x * | Deadlift | 90% | 5x * |
| Deadlift | 80% | 3x * | Squat | 80% | 3x * | Bench | 80% | 3x * |
| RFESS | 75% | 3x8 | Low-incline Bench | 75% | 3x8 | OHP | 75% | 3x8 |
| RDL | 75% | 3x8 | Lat Pulldown | 75% | 3x8 | BB Row | 75% | 3x8 |
| Face-pull | 75% | 3x8 | Goblet Squat | 75% | 3x8 | BB curl | 75% | 3x8 |
| Skull Crushers | 75% | 3x8 |  |  |  | Lat Raises | 75% | 3x8 |
| Week 5 | | | | | | | | |
| Day 13 | | | Day 14 | | | Day 15 | | |
| Exercise | Weight | SxR | Exercise | Weight | SxR | Exercise | Weight | SxR |
| Squat | 95% | 6x * | Bench | 95% | 6x * | Deadlift | 95% | 6x * |
| Deadlift | 85% | 3x * | Squat | 85% | 3x * | Bench | 85% | 3x * |
| RFESS | 80% | 3x6 | Low-incline Bench | 80% | 3x6 | OHP | 80% | 3x6 |
| RDL | 80% | 3x6 | Lat Pulldown | 80% | 3x6 | BB Row | 80% | 3x6 |
| Face-pull | 80% | 3x6 | Goblet Squat | 80% | 3x6 | BB curl | 80% | 3x6 |
| Skull Crushers | 80% | 3x6 |  |  |  | Lat Raises | 80% | 3x6 |
| Week 6 | | | | | | | | |
| Day 16 | | | Day 17 | | | Day 18 | |  |
| Exercise | Weight | SxR | Exercise | Weight | SxR | Exercise | Note |  |
| Squat | 65% | 3x3 | Squat | 65% | 3x3 | Squat | Strength testing for these variables |  |
| Deadlift | 65% | 3x3 | Deadlift | 65% | 3x3 | Deadlift |  |  |
| Bench | 65% | 3x3 | Bench | 65% | 3x3 | Bench |  |  |
|  |  |  |  |  |  |  |  |  |
